# The *NF-Y* gene family in *Rosa*: evolutionary divergence across three species and potential roles in heat tolerance and secondary metabolism

**DOI:** 10.3389/fpls.2026.1944775

**Published:** 2026-10-08

**Authors:** Sainan Jiang, Jun Zhang, Jie Yang, Junlan Zhang, Wen Xing, Zeqing Li

**Affiliations:** 1 School of Pharmacy, Hubei University of Science and Technology, Xianning, China; 2 Key Laboratory of National Forestry and Grassland Administration on Osmanthus fragrans, Hubei University of Science and Technology, Xianning, China; 3 College of Landscape Architecture, Central South University of Forestry and Technology, Changsha, China

**Keywords:** *Rosa*, NF-Y transcription factor, secondary metabolism, heat stress response, transcriptional regulation, multi-omics analysis

## Abstract

**Introduction:**

Plant pigmentation and secondary metabolism are intrinsically linked to pollinator attraction, environmental adaptation, and ornamental quality, yet the transcriptional regulators that coordinate multiple metabolic branches remain poorly understood. Nuclear Factor Y (NF-Y) transcription factors are well-established mediators of developmental and stress signaling, but their involvement in orchestrating plant metabolic networks has not been explored in the genus *Rosa *.

**Methods:**

Here, we performed genome-wide identification and multi-omics characterization of 30, 30, and 29 *NF-Y* genes from *R. chinensis*, *R. rugosa*, and *R. wichuraiana*, respectively. We then conducted evolutionary analyses, orthology-based protein interaction modeling, promoter cis-element analysis, transcriptome profiling under heat stress, and integrated Weighted Gene Co-expression Network Analysis (WGCNA) with untargeted metabolomics.

**Results:**

Evolutionary analyses revealed that segmental duplications drove family expansion under strong purifying selection, with the *NF-YB * and *NF-YC* subfamilies exhibiting distinct structural diversification. Orthology-based protein interaction modeling predicted the NF-YC subgroup I as a likely scaffold for heterotrimer assembly, while promoter cis-element analysis showed enrichment in hormone-responsive motifs (ABRE and MeJA), linking these genes to both environmental cues and metabolic signaling. Transcriptome profiling under heat stress demonstrated species-specific *NF-Y* expression dynamics, with the *NF-YA* subfamily contributing the most responsive members. Crucially, by integrating WGCNA with untargeted metabolomics, we associated multiple *RcNF-Y* genes with cultivar-dependent accumulation of flavonoids, terpenoids, and alkaloids—spanning the major classes of pigmentation-related and defense-related secondary metabolites. Among these, *RcNF-YA4, RcNF-YA5*, and *RcNF-YB12* were associated with flavonoid accumulation, whereas *RcNF-YC2* was associated with terpenoid and alkaloid accumulation. This suggests that *RcNF-YC2* may function as a potential hub bridging stress signaling with broad-spectrum metabolic regulation. Notably, the *NF-Y * members responding to heat stress and those linked to metabolic output showed limited overlap, implying functional specialization within the family.

**Discussion:**

Collectively, our findings suggest a potential role of the rose *NF-Y * family in integrating thermal stress signals with diverse secondary metabolic pathways. This study prioritizes *RcNF-YC2* as a candidate target for genetic or biotechnological improvement of fragrance and stress resilience in ornamental crops.

## 1 Introduction

NF-Y is a ubiquitous heterotrimeric transcription factor in eukaryotes, typically composed of three distinct subunits: NF-YA (synonyms HAP2 or CBF-B), NF-YB (HAP3 or CBF-A), and NF-YC (HAP5 or CBF-C). Its functional activity depends on the dimerization of NF-YB and NF-YC in the cytoplasm, followed by their translocation into the nucleus where they recruit NF-YA to assemble an active ternary complex. This complex then specifically recognizes and binds to the CCAAT-box element in the promoters of downstream target genes to regulate transcription (Mantovani, 1999; Hackenberg et al., 2012). In addition, some studies have also shown that NF-YB/NF-YC dimers can bypass NF-YA members and form heterotrimers with other transcription factors (Zhao et al., 2017; Myers and Holt, 2018). Unlike in yeast and mammals, where each subunit is encoded by only one or two genes, the *NF-Y* gene family has undergone significant expansion in plants. For example, *Arabidopsis thaliana* contains 36 members (10 *NF-YA*, 13 *NF-YB*, 13 *NF-YC*) (Siefers et al., 2009), *Oryza sativa* has 40 members (Manimaran et al., 2017), and *Solanum lycopersicum* has as many as 59 members (Li et al., 2016). This expansion endows plants with a tremendous combinatorial diversity in NF-Y complex assembly, enabling the fine-tuned regulation of various complex biological processes.

Numerous studies have demonstrated that plant NF-Y transcription factors are extensively involved in regulating growth, development, and stress responses. In terms of growth and development, *NF-Y* family members play critical roles in embryogenesis (Mu et al., 2013), seed development (Kwong et al., 2003), flowering time regulation (Kumimoto et al., 2010; Hou et al., 2014), and root elongation (Ballif et al., 2011). For instance, *LEC1* (*AtNF-YB9*) and its homologs in Arabidopsis are key regulators of embryogenesis and seed maturation (Kwong et al., 2003; Lee et al., 2003). Regarding stress responses, *NF-Y* genes have been shown to participate in adaptive responses to various abiotic stresses, including drought (Nelson et al., 2007; Li et al., 2008), salinity (Chen et al., 2015; Ma et al., 2015), low temperature (Shi et al., 2014), and high temperature (Rao et al., 2022; Wang et al., 2023). For example, overexpression of *AtNF-YB1* significantly enhances drought tolerance in Arabidopsis (Nelson et al., 2007); overexpression of *ZmNF-YB16* improves drought tolerance in maize by enhancing photosynthesis and antioxidant capacity (Wang et al., 2018); and in kiwifruit, *AcNF-YC22* has been confirmed to positively regulate heat stress tolerance by protecting the photosynthetic system from heat damage (Zhang et al., 2026). Furthermore, the expression of *NF-Y* genes is induced by various phytohormones, such as abscisic acid (ABA) and methyl jasmonate (MeJA), suggesting that they act as critical downstream nodes in signaling pathways, integrating hormonal signals with stress responses (Li et al., 2008; Hwang et al., 2019).

In addition to their roles in stress adaptation, recent studies have revealed novel functions of *NF-Y* in regulating plant secondary metabolism. In tomato, a complex formed by NF-YB8, NF-YC1, and NF-YA1/9 directly binds to the CCAAT element in the *CHS1* promoter, affecting flavonoid biosynthesis by modulating H3K27me3 levels and thereby determining peel color (Wang et al., 2021). Additionally, SlNF-YA10 has been reported to negatively regulate ascorbic acid (AsA) accumulation by binding to the promoters of *GME* and *GGP* (Chen et al., 2020). As a major antioxidant, AsA is closely associated with plant stress tolerance, but this function is distinct from the transcriptional regulation of secondary metabolic pathways (Alayafi, 2020). These findings indicate that the *NF-Y* family possesses multifaceted functions, participating independently in both stress responses and metabolic regulation.

Plants of the genus *Rosa* are globally important ornamentals, with *R. chinensis*, *R. rugosa*, and *R. wichuraiana* being the main progenitors of modern garden roses, possessing high economic and cultural value (Hibrand Saint-Oyant et al., 2018; Raymond et al., 2018; Cui et al., 2020; Chen et al., 2021). However, the production and ornamental quality of *Rosa* plants are often constrained by high-temperature stress, and flower color and fragrance is a key trait determining their ornamental value. Previous studies have shown that high-temperature stress significantly affects the accumulation of anthocyanins and volatile terpenoids in rose petals, thereby influencing flower color and fragrance (Dela et al., 2003; Zhang et al., 2025). Nevertheless, these are separate biological processes—stress physiology and metabolic biochemistry—and whether *NF-Y* genes in *Rosa* participate in either or both remains completely unexplored.

Although the *NF-Y* gene family has been systematically characterized in many plants, its genome-wide features, evolutionary dynamics, and potential functions in heat stress response and secondary metabolic regulation in *Rosa* remain unknown. In this study, based on the published genome data of the three *Rosa* species, we systematically identified and compared the *NF-Y* gene family at the genome-wide level. We first analyzed the sequence characteristics, phylogenetic relationships, duplication events, gene structures, and conserved motifs of this family. Subsequently, we investigated the potential functions and expression divergence patterns of *NF-Y* genes through promoter analysis, tissue-specific expression profiling, and heat stress response analysis. Finally, using weighted gene co-expression network analysis (WGCNA) combined with metabolomics, we predicted key *NF-Y* genes involved in the biosynthesis of secondary metabolites, particularly those related to different color varieties. The results of this study will provide important theoretical foundations and genetic resources for a deeper understanding of the evolution and function of *NF-Y* genes in *Rosa*, as well as for the genetic improvement of stress tolerance and flower color in *Rosa* species.

## 2 Materials and methods

### 2.1 Plant materials and experimental design

#### 2.1.1 Plant materials for transcriptome and metabolome analyses

A total of two *R. chinensis* and eight *R. hybrida* cultivars were used for untargeted metabolomics analysis: white-colored (‘Bolero’, abbreviated as BLLW; ‘Pope John Paul II’, PLII), red-colored (‘Black Baccara’, HBK; ‘Huo Linglong’, HLL; ‘Slater’s crimson China’, YYH; ‘Memorial Barbara’, JNBBL), yellow-colored (‘Canary’, JSQ), orange-colored (‘Masora’, ZZ), and pink-colored (‘Xizi Yanyu’, XZYY; ‘Old Blush’, YYF). Among these, six cultivars (BLLW, PLII, HBK, HLL, YYH, JSQ) were also subjected to RNA-seq. All plants were two-year-old healthy hardwood cuttings, grown under natural outdoor conditions at the experimental field of Central South University of Forestry and Technology in Changsha, Hunan Province, China (28°06′ N, 113°01′ E). The local climate is characterized by a humid subtropical monsoon climate with a mean annual temperature of approximately 17.4 °C and mean annual precipitation of about 1,500 mm. During the experimental period (April to May), the average daytime temperature was approximately 22–26 °C with natural photoperiod (approximately 13 h light/11 h dark) and relative humidity ranging from 70% to 85%. Petals at full expansion stage were collected from all cultivars, immediately frozen in liquid nitrogen, and stored at −80 °C until use. All samples were collected between 8:00 and 9:00 AM to minimize diurnal variation in metabolite and transcript profiles.

#### 2.1.2 Publicly available transcriptomic data

Tissue-specific expression profiles of *NF-Y* genes in three *Rosa* species were obtained from public databases: *R. chinensis* cv. ‘Old Blush’: six tissues (root, stem, leaf, prickle, stamen, pistil/ovary); *R. rugosa*: five tissues (root, leaf, stamen, petal, fruit); *R. wichuraiana*: four tissues (root, stem, leaf, bud). All public datasets were retrieved from the NCBI Sequence Read Archive (SRA) under accession numbers listed in Data Availability. For heat stress responses, leaf transcriptomes of *R. chinensis* cv. ‘Slater’s Crimson China’ and *R. rugosa* cv. ‘BaoWhite’ were downloaded from our previous studies (Li ZQ. et al., 2019; Ding et al., 2024), in which samples were collected under control (0 h) and heat treatment conditions—42 °C for 2 h for *R. chinensis* and 45 °C for 2 h for *R. rugosa*—with three biological replicates per condition.

### 2.2 Gene identification, physicochemical properties and subcellular localization prediction

The genome assembly and annotation of *R. chinensis* were downloaded from the NCBI Genome database under accession number GCF_002994745.2. Genomes of *R. rugosa* (v1.0) and *R. wichuraiana* (v1.0) were retrieved from the Genome Database for Rosaceae (GDR, https://www.rosaceae.org/). Genomes of *A*. *thaliana* (TAIR10) and *O. sativa* (MSU7) were obtained from their respective public repositories. To identify *NF-Y* family members, an integrated BLASTP and HMMER approach was employed. BLASTP searches were performed using 36 *A*. *thaliana* and 40 *O. sativa* NF-Y full-length protein sequences retrieved from PlantTFDB as queries and list in **Supplementary Table S1**. Concurrently, HMMER v3.2.1 was used with the hidden Markov models of conserved NF-Y domains (PF00808, PF02045). The conserved domains of candidate genes were validated using PFAM, CDD, and SMART databases. Physicochemical properties (amino acid length, molecular weight, theoretical pI, instability index, aliphatic index, grand average of hydropathicity (GRAVY)) were predicted with ExPASy ProtParam (https://web.expasy.org/protparam/). Subcellular localization was predicted using WoLF PSORT (https://wolfpsort.hgc.jp/). Sequence similarities were calculated using Tbtools with default parameters (Chen et al., 2023).

### 2.3 Phylogenetic analysis

NF-Y protein sequences from the three *Rosa* species, *A. thaliana*, and *O. sativa* were aligned using MUSCLE v3.8.31 with default settings (Edgar, 2004). Phylogenetic trees were constructed using the neighbor-joining (NJ) method implemented in MEGA11 v11.0.13, with the 1000 bootstrap replicates (Tamura et al., 2021). Trees were visualized using iTOL (https://itol.embl.de/).

### 2.4 Synteny analysis and selection pressure assessment

Intra-species, inter-species, and cross-species synteny analyses were performed using MCScanX with default parameters (Wang et al., 2024). Duplicated gene pairs were identified based on genomic coordinates. The non-synonymous (Ka) and synonymous (Ks) substitution rates were calculated using KaKs_Calculator v2.0 with the NG (Nei-Gojobori) method. Pairs with Ka/Ks ratios were classified as undergoing purifying selection (Ka/Ks < 1), neutral evolution (Ka/Ks = 1), or positive selection (Ka/Ks > 1). Syntenic relationships were visualized using TBtools and Circos.

### 2.5 Conserved domains, motifs, and gene structure analysis

Conserved domains were identified using NCBI CDD (v3.19) and SMART (v8.0) with an E-value threshold of 0.01. Motif analysis was performed with MEME Suite v5.5.8 (motif width 6–50 aa, maximum 10 motifs). Gene structures (exon-intron organization, UTRs) were extracted from GFF annotation files and visualized using TBtools.

### 2.6 Protein-protein interaction network prediction

PPI networks were predicted based on orthology to *A. thaliana* using the STRING database v12.0 with confidence score ≥ 0.4 (https://cn.string-db.org/). Networks were constructed and visualized in Cytoscape v3.10.0 (Su et al., 2014). Core proteins were defined as those with a degree (number of interactions) greater than half of the total nodes in the network.

### 2.7 Promoter cis-acting element analysis

The 2 kb promoter sequences upstream of the start codon for each *NF-Y* gene were extracted from the genome assemblies. Cis-acting elements were identified using PlantCARE with default parameters. Elements were classified into four categories: light-responsive, phytohormone-responsive, plant growth and development, and stress-responsive. Distribution maps were generated using TBtools and R package ggplot2.

### 2.8 Processing of public transcriptomic data

Raw RNA-seq reads from public sources were quality-trimmed using Trimmomatic (v0.39) and aligned to the respective reference genomes using HISAT2 v2.2.1 (Kim et al., 2019). Gene expression levels were quantified as FPKM using featureCounts and a custom R script. The tissue-specific expression patterns of *RrNF-Y* genes were retrieved from the FPKM matrix reported by Wei et al. (2024). For differential expression analysis under heat stress, DESeq2 v1.36.0 was applied with thresholds |log_2_FoldChange| ≥ 1 and adjusted p-value (Benjamini-Hochberg) < 0.05. Heatmaps were generated with the Tbtools.

### 2.9 RNA-seq of roses and WGCNA

#### 2.9.1 RNA extraction, library preparation, and sequencing

For the six cultivars, total RNA was extracted from petal tissues using the RNAprep Pure Plant Kit (Tiangen, Beijing, China). RNA quality and integrity were assessed using a Qubit3.0 (Thermo Fisher, USA) and a Qsep400 instrument (Bioptic, China). mRNA was enriched using oligo(dT) magnetic beads (NEB, USA). Libraries were constructed using the NEBNext Ultra RNA Library Prep Kit (NEB) according to the manufacturer’s protocol and sequenced on the Illumina NovaSeq 6000 platform (PE150) by Wuhan IGENEBOOK Biotechnology Co.,Ltd (http://www.igenebook.com). Three biological replicates were sequenced for each cultivar.

#### 2.9.2 WGCNA

Raw reads were processed as described in Section 2.8. To enrich for genes with coordinated expression changes relevant to flower color and secondary metabolism, the co-expression network was constructed using the union of differentially expressed genes (DEGs) identified between colored and white cultivars (|log_2_FC| ≥ 1, adjusted p < 0.05). The R package WGCNA was used to construct co-expression networks (Langfelder and Horvath, 2008). A soft-thresholding power β (β = 14) was selected to achieve scale-free topology. Modules were identified with a minimum module size of 30 genes and a cut height of 0.25. Module-trait associations were assessed by correlating module eigengenes with cultivar traits using Pearson correlation. KEGG enrichment analysis for genes in modules containing *RcNF-Y* members was performed using clusterProfiler with adjusted p-value < 0.05 (Wu et al., 2021).

### 2.10 Untargeted metabolomics of roses

#### 2.10.1 Metabolite extraction and UPLC-MS/MS analysis

Petal tissues from the 10 cultivars were collected and freeze-dried. One biological sample (a single plant) was collected per cultivar; because this was an exploratory profiling study of the metabolite landscape across cultivars and color groups, independent cultivars within each color group served as the biological units for group comparison. Metabolites were extracted using 70% methanol containing internal standards. Untargeted metabolomics analysis was performed using an ultra-high-performance liquid chromatography system coupled with tandem mass spectrometry (UPLC-MS/MS). Chromatographic separation was achieved on Waters ACQUITY UPLC HSS T3 column (2.1 × 100 mm, 1.8 μm) with a gradient of water/acetonitrile (both containing 0.1% formic acid). Mass spectra were acquired in positive and negative ion modes. Quality control (QC) samples were prepared by pooling equal aliquots of all samples and were injected periodically (every 10 samples) to monitor instrument stability.

#### 2.10.2 Data processing and metabolite identification

Raw MS data were processed using XCMS (v3.12.0) for peak detection, alignment, and retention-time correction. Features with >50% missing values across all samples were excluded. For the remaining features, missing values were imputed using a hybrid strategy: features with a missing rate >50% were assigned one-fifth of the minimum observed value, whereas those with ≤50% missingness were filled via the k-nearest neighbors (KNN) algorithm. Peak areas were then corrected using support vector regression (SVR) with a radial basis function kernel, referencing quality control (QC) samples to minimize batch-to-batch variation. Metabolite annotation was performed against an in-house spectral library, public databases (HMDB, MassBank, and METLIN), in-silico prediction libraries, and the metDNA algorithm. Putative identifications with a comprehensive confidence score ≥0.5 were retained, and only metabolites with a QC coefficient of variation (CV) <0.5 were kept. Positive- and negative-ion mode data were merged; for metabolites detected in both modes, the entry with the highest confidence tier (and, secondarily, the highest composite score) was selected as the final representative. To identify differentially accumulated metabolites (DAMs) associated with color-group differentiation, univariate analyses were performed comparing colored accessions (red, pink, and yellow/orange) against white cultivars. Candidate DAMs were defined as metabolites satisfying both P < 0.05 and |log_2_(Fold change)| ≥ 1.

### 2.11 Correlation network construction

Pearson correlation coefficients were calculated among (i) the expression levels of *RcNF-Y* genes, (ii) the abundance of flavonoids, terpenoids, and alkaloids, and (iii) the expression of key biosynthetic genes, using the six cultivars as matched observations (n = 6). For each cultivar, the transcriptome values were averaged across the three biological RNA-seq replicates, whereas the metabolome was represented by a single sample per cultivar; the two data layers were therefore biologically matched at the cultivar level. The strong correlation between *RcNF-Y* and structural genes (|r| > 0.9, P < 0.01) and structural genes and metabolites (|r| > 0.8, P < 0.05) were retained to construct the network using Cytoscape v3.10.0.

## 3 Results

### 3.1 Genome-wide identification and basic characterization of *NF-Y* genes

To systematically identify NF-Y transcription factors in *Rosa* species, an integrated BLASTP and HMMER approach was employed. BLASTP initially identified 30, 31, and 31 candidate *NF-Y* genes in *R. chinensis*, *R. rugosa*, and *R. wichuraiana* (designated as *RcNF-Y*, *RrNF-Y*, and *RwNF-Y*, respectively), whereas HMMER yielded 32, 32, and 30 candidates, respectively. After verification of conserved domains, a final set of 30 *RcNF-Y*, 30 *RrNF-Y*, and 29 *RwNF-Y* genes was confirmed; this set comprises canonical *NF-Y* subunits together with divergent NF-Y-like members carrying NC2/DPB-related histone-fold domains. According to subfamily classification, *RcNF-Y* comprised 7 *NF-YA*, 13 *NF-YB*, and 10 *NF-YC* members; *RrNF-Y* contained 6 *NF-YA*, 14 *NF-YB*, and 10 *NF-YC* members; and *RwNF-Y* consisted of 4 *NF-YA*, 15 *NF-YB*, and 10 *NF-YC* members (**Supplementary Table S2**).

Basic physicochemical properties of the identified NF-Y proteins, including amino acid length, molecular weight, theoretical isoelectric point (pI), instability index, aliphatic index, grand average of hydropathicity (GRAVY), and subcellular localization, were further analyzed. The results revealed subfamily-specific physicochemical features (**Supplementary Table S3** and **S4**). NF-YA subfamily members exhibited the largest average amino acid length and molecular weight, whereas NF-YB members showed the smallest. Regarding theoretical pI, all NF-YA members from the three species had pI values > 7.0, indicating basic proteins; in contrast, only one and two basic proteins were identified in the NF-YB and NF-YC subfamilies, respectively. Instability index analysis showed that only three NF-YB proteins had indices < 40, classifying them as stable proteins. The aliphatic index was highest in the NF-YC subfamily and lowest in NF-YA across the three species, predicted that NF-YC may possess better thermal stability, whereas NF-YA may be relatively thermolabile. All target proteins had GRAVY values < 0, indicating hydrophilic nature; however, NF-YA proteins exhibited significantly stronger hydrophilicity than NF-YB and NF-YC proteins. Subcellular localization prediction revealed that all NF-YA proteins were localized in the nucleus; NF-YB proteins were mainly distributed in the nucleus and mitochondria, with only one member each localized to the chloroplast; NF-YC proteins were distributed in the nucleus and chloroplast, with only one member each localized to the cytoplasm.

To further evaluate the evolutionary relationships among *Rosa* NF-Y proteins, sequence similarities were analyzed both within and between species. The results showed that, irrespective of intra- or interspecies comparisons, members of the same subfamily exhibited significantly higher similarity than members from different subfamilies, particularly within the NF-YA and NF-YB subfamilies (**Figure 1**; **Supplementary Figure 1**; **Supplementary Table S5**). Across different subfamilies, the similarity between NF-YA and NF-YB or NF-YC was lower than that between NF-YB and NF-YC (**Supplementary Figure 1**; **Supplementary Table S5**), consistent with the shared conserved domain features between NF-YB and NF-YC. Collectively, sequence divergence may serve as the structural basis for functional differentiation among *Rosa* NF-Y subfamilies; gene duplication events occurred within subfamilies, and the *NF-Y* genes from the three *Rosa* species exhibited co-evolutionary features.

**Figure 1:**
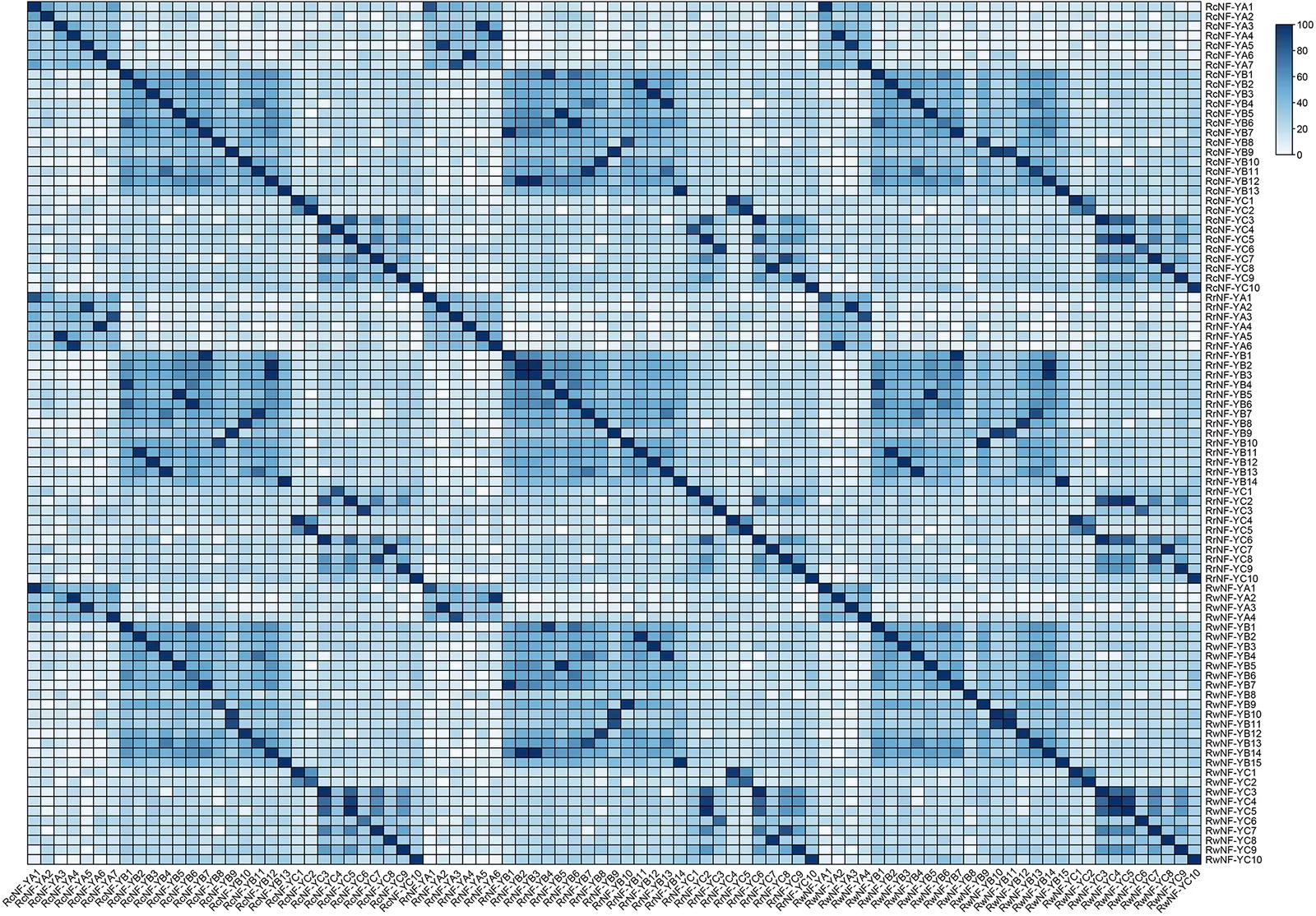
Heatmap of amino acid sequence similarity among *Rosa* NF-Y proteins. Colored blocks indicate the percentage similarity between two proteins, with darker blue representing higher similarity.

### 3.2 Phylogenetic relationships and subgroup classification of NF-Y proteins

To resolve the phylogenetic relationships of NF-Y proteins from the three *Rosa* species, a phylogenetic tree was constructed using the newly identified RcNF-Y, RrNF-Y, and RwNF-Y protein sequences along with known NF-Y sequences from *A. thaliana* and *O. sativa*, totaling 165 proteins. The results demonstrated that all NF-Y proteins were clearly clustered into subfamilies (NF-YA, NF-YB, and NF-YC), and the phylogenetic distance between NF-YB and NF-YC was significantly closer than their distances to NF-YA, which was highly consistent with the sequence similarity analysis described above (**Figure 2A**). Further observation revealed that within each subfamily, small branches could be distinctly classified into *Rosa*-derived and non-*Rosa*-derived groups, indicating that *Rosa NF-Y* genes have evolved into relatively independent lineages.

**Figure 2:**
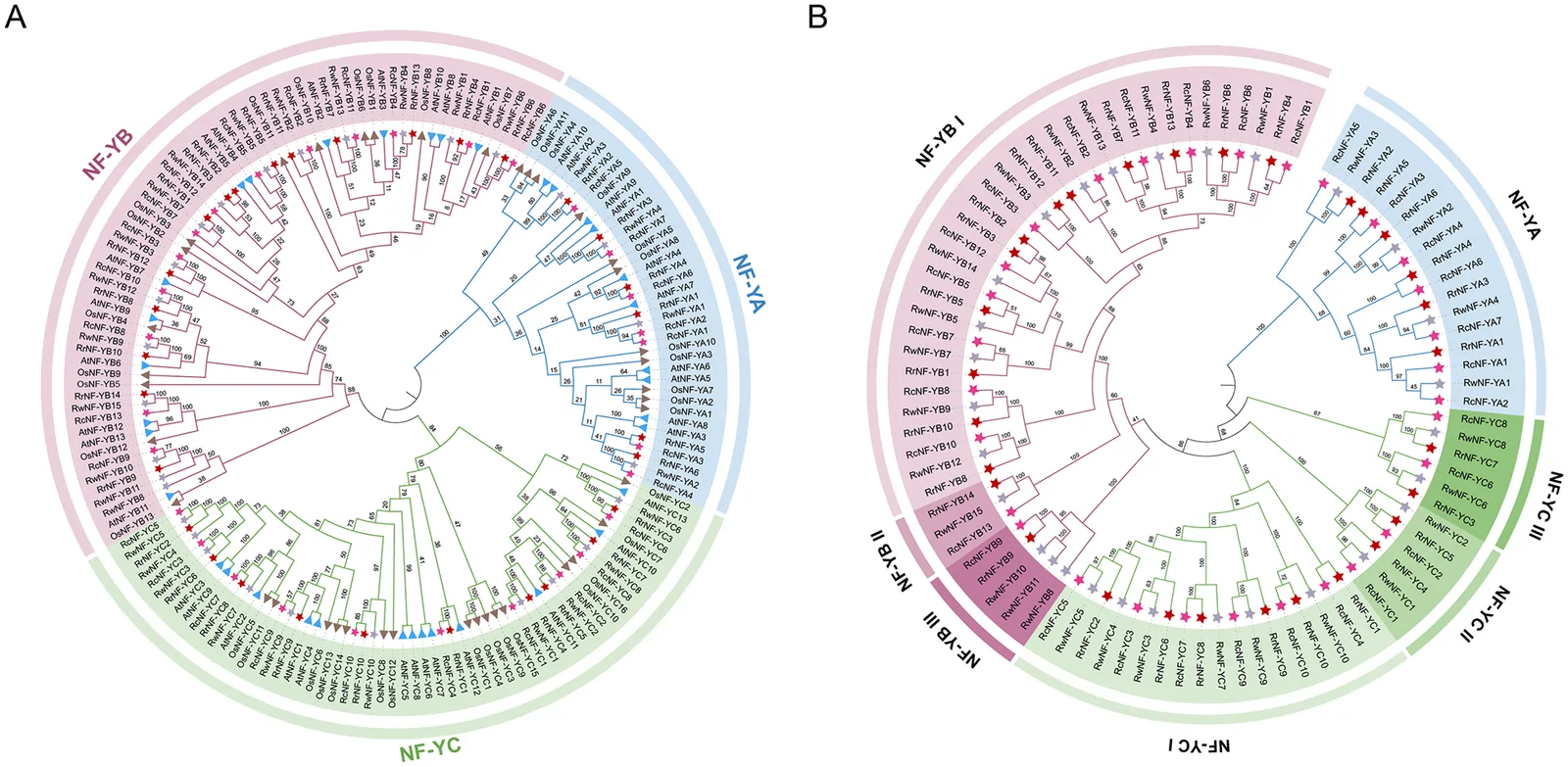
Phylogenetic trees of NF-Y proteins from *Rosa* and other plants **(A)** and from *Rosa* alone **(B)**. Proteins from *Rosa*, *A. thaliana*, and *O. sativa* are indicated by differently colored stars, left-pointing triangles, and right-pointing triangles, respectively. Subfamilies and subgroups are marked with color-coded classification bars.

To deeply investigate the evolutionary structure within *Rosa*, a separate phylogenetic tree was reconstructed using only the NF-Y protein sequences from the three *Rosa* species (**Figure 2B**). The results showed that the NF-YB and NF-YC subfamilies could each be further divided into three subgroups. Specifically, NF-YB subgroup I contained 34 proteins; except for one branch (RcNF-YB12/RrNF-YB2/RrNF-YB3/RwNF-YB14) that contained an additional RrNF-YB member, all other branches consisted of one NF-YB member from each of the three species. NF-YB subgroup II comprised RcNF-YB13, RrNF-YB14, and RwNF-YB15. NF-YB subgroup III consisted of RcNF-YB9, RrNF-YB9, RwNF-YB8, RwNF-YB10, and RwNF-YB11. For the NF-YC subfamily, subgroups I, II, and III contained six, two, and two NF-YC members from each species, respectively. Within NF-YC subgroup I, one branch (RcNF-YC4/RrNF-YC1) lacked the corresponding RwNF-YC member, whereas another branch (RcNF-YC5/RrNF-YC2/RwNF-YC4/RwNF-YC5) contained two RwNF-YC members (RwNF-YC4 and RwNF-YC5).

In summary, the phylogenetic tree of *Rosa* NF-Y proteins not only supported the evolutionary proximity between the NF-YB and NF-YC subfamilies but also revealed further diversification within each subfamily. The uneven distribution of members among subgroups and the presence of species-specific gene duplication or loss events in certain branches suggest that *Rosa NF-Y* genes underwent lineage-specific evolution after species divergence.

### 3.3 Synteny analysis and selection pressure assessment of *NF-Y* genes

To investigate duplication events and evolutionary driving forces of *Rosa NF-Y* genes, intra-species, inter-species, and cross-species synteny analyses were performed, and the ratios of non-synonymous (Ka) to synonymous (Ks) substitution rates (Ka/Ks) were calculated for duplicated gene pairs. Intra-species synteny analysis revealed several syntenic gene pairs in the genomes of *R. chinensis*, *R. rugosa*, and *R. wichuraiana*, which may have originated from segmental duplication or whole-genome duplication events (**Figures 3A–C**; **Supplementary Table S6**). Specifically, *R. chinensis* contained one *NF-YA* duplicated pair (*RcNF-YA3*/*RcNF-YA4*) and two *NF-YB* duplicated pairs (*RcNF-YB1*/*RcNF-YB6* and *RcNF-YB5*/*RcNF-YB7*); *R. rugosa* contained one *NF-YA* duplicated pair (*RrNF-YA5*/*RrNF-YA6*) and three *NF-YB* duplicated pairs (*RrNF-YB2*/*RrNF-YB3*, *RrNF-YB4*/*RrNF-YB6*, and *RrNF-YB10*/*RrNF-YB13*); *R. wichuraiana* contained three *NF-YB* duplicated pairs (*RwNF-YB1*/*RwNF-YB6*, *RwNF-YB5*/*RwNF-YB7*, and *RwNF-YB7*/*RwNF-YB14*) and one *NF-YC* duplicated pair (*RwNF-YC3*/*RwNF-YC5*). Ka/Ks values for these duplicated pairs, excluding those for which Ks could not be calculated, were all less than 1, indicating that these genes have been mainly subjected to purifying selection following intra-species duplication (**Supplementary Table S6**).

**Figure 3:**
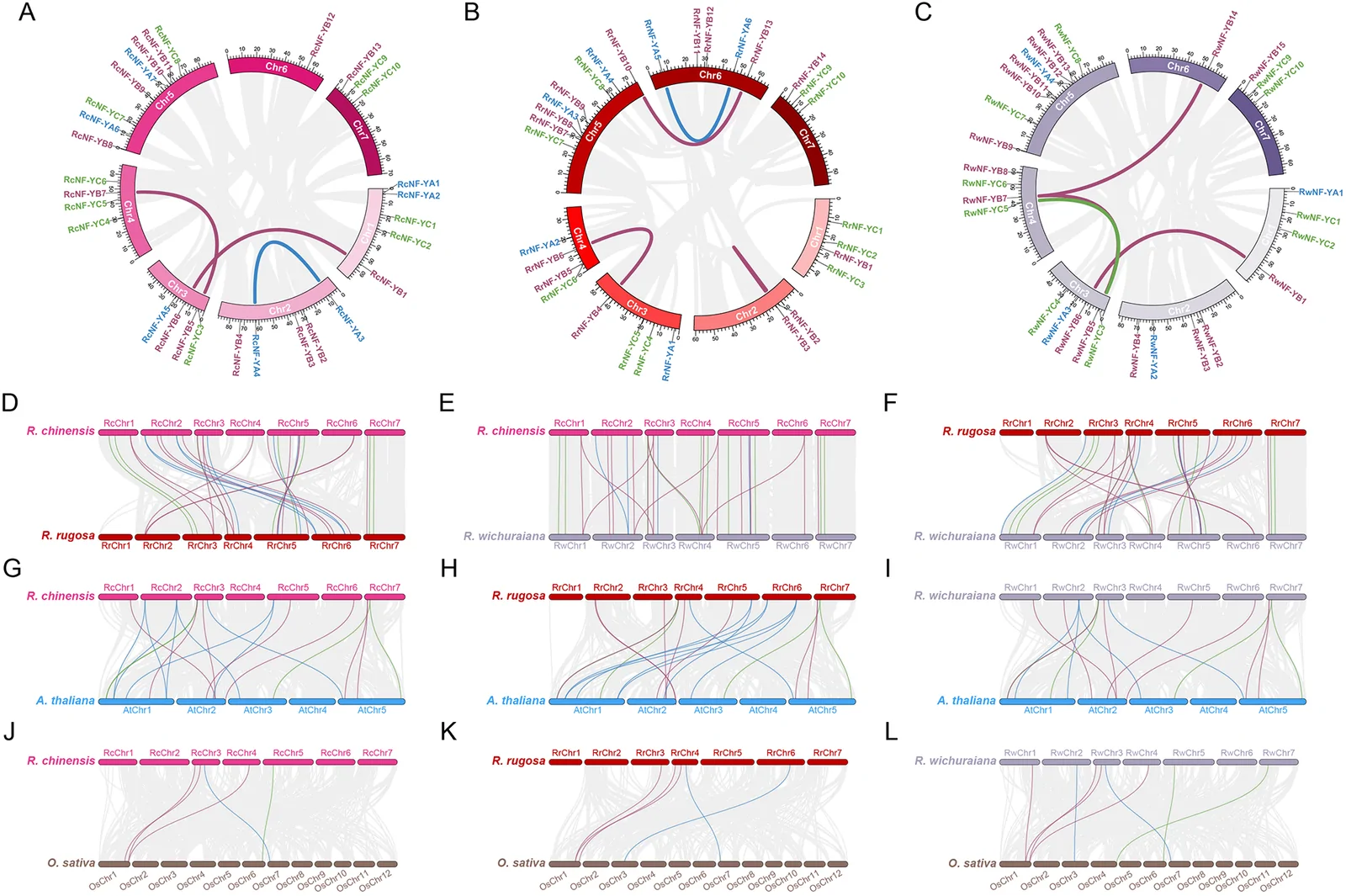
Synteny analysis of *Rosa NF-Y* genes. **(A–C)** Intra-species synteny in *R. chinensis* **(A)**, *R. rugosa* **(B)**, and *R. wichuraiana* **(C)**. **(D–F)** Inter-species synteny between *R. chinensis* and *R. rugosa* **(D)**, *R. chinensis* and *R. wichuraiana* **(E)**, and *R. rugosa* and *R. wichuraiana* **(F)**. **(G–I)** Synteny between *R. chinensis* **(G)**, *R. rugosa* **(H)**, and *R. wichuraiana* **(I)** with *A. thaliana*. **(J–L)** Synteny between *R. chinensis* **(J)**, *R. rugosa* **(K)**, and *R. wichuraiana* **(L)** with *O. sativa*.

Inter-species synteny analysis showed abundant syntenic relationships among the three *Rosa* species (**Figures 3D–F**; **Supplementary Table S6**). Between *R. chinensis* and *R. rugosa*, a total of 30 syntenic gene pairs were identified, including seven *NF-YA* pairs (involving five *RcNF-YA* and five *RrNF-YA* genes), 16 *NF-YB* pairs (involving 13 *RcNF-YB* and 13 *RrNF-YB* genes), and seven *NF-YC* pairs (involving seven *RcNF-YC* and seven *RrNF-YC* genes). Between *R. chinensis* and *R. wichuraiana*, 32 pairs were identified, including four *NF-YA* pairs (involving four *RcNF-YA* and three *RwNF-YA* genes), 18 *NF-YB* pairs (involving 13 *RcNF-YB* and 13 *RwNF-YB* genes), and 10 *NF-YC* pairs (involving nine *RcNF-YC* and nine *RwNF-YC* genes). Between *R. rugosa* and *R. wichuraiana*, 31 pairs were identified, including five *NF-YA* pairs (involving five *RrNF-YA* and four *RwNF-YA* genes), 18 *NF-YB* pairs (involving 13 *RrNF-YB* and 13 *RwNF-YB* genes), and eight *NF-YC* pairs (involving seven *RrNF-YC* and eight *RwNF-YC* genes). For these orthologous pairs, except for a few cases where Ks could not be calculated or Ka/Ks was marginally > 1, the vast majority had Ka/Ks values < 1, suggesting that orthologous genes in *Rosa* have also been predominantly shaped by purifying selection since species divergence (**Supplementary Table S6**).

To further assess the evolutionary conservation of *Rosa NF-Y* genes on a broader evolutionary scale, syntenic relationships between the three *Rosa* species and the monocot *O. sativa* as well as the dicot *A. thaliana* were analyzed. The number of syntenic pairs between each *Rosa* species and *O. sativa* (five for *R. chinensis*, five for *R. rugosa*, and eight for *R. wichuraiana*) was significantly lower than that with *A. thaliana* (19, 20, and 16, respectively) (**Figures 3G–L**; **Supplementary Table S6**). Notably, within the *NF-YA* subfamily, eight syntenic relationships were identified between *R. chinensis* and *A. thaliana* and between *R. rugosa* and *A. thaliana*, involving four *RcNF-YA* and four *RrNF-YA* genes, respectively. Taken together, these results indicate that *Rosa NF-Y* genes have been highly conserved during evolution; the *NF-YA* subfamily tends to be inherited and retained from dicot ancestors, whereas expansion of the *NF-YB* subfamily may be closely associated with whole-genome or segmental duplication events.

### 3.4 Domain, conserved motif, and gene structure characterization of NF-Y proteins

To elucidate the structural basis of *Rosa* NF-Y protein function, domain composition, conserved motif distribution, and gene structure features were analyzed. Domain analysis revealed that the domain composition of *Rosa* NF-Y proteins exhibited clear subfamily- and subgroup-specific characteristics, being highly consistent within the same subfamily or subgroup but differing among subgroups (**Figures 4A, C**). Specifically, all NF-YA members contained a CBF or CBFB_NFYA domain, with RcNF-YA5 additionally containing a HAP2 superfamily domain. In the NF-YB subfamily, all members of subgroup I carried the HFD_NFYB domain, whereas subgroups II and III harbored the HFD_Dr1 and HFD_POLE3_DPB4 domains, respectively. The NF-YC subfamily showed more complex differentiation: all members of subgroup I contained an HFD_NFYC-like domain, with RrNF-YC1 additionally containing a HAP5 superfamily domain; all members of subgroup II contained the HFD_DRAP1 domain; subgroup III displayed two branches—the branch (RcNF-YC6/RrNF-YC3/RwNF-YC6) contained the HFD_POLE4-like domain, whereas the other branch (RcNF-YC8/RrNF-YC7/RwNF-YC8) carried the HFD_SF superfamily domain, with RrNF-YC7 additionally containing a histone superfamily domain. Notably, the HFD_Dr1, HFD_DRAP1, HFD_POLE3_DPB4, and HFD_POLE4-like domains are the histone-fold domains of the NC2β (Dr1), NC2α (DRAP1), DPB4 (POLE3), and POLE4 proteins, respectively, which belong to histone-fold protein families distinct from canonical NF-Y. Members carrying these domains were therefore classified as divergent NF-Y-like proteins, whereas the canonical NF-Y subunits correspond to the NF-YA subfamily and to the NF-YB/NF-YC subgroup I members carrying the HFD_NFYB/HFD_NFYC domains.

**Figure 4:**
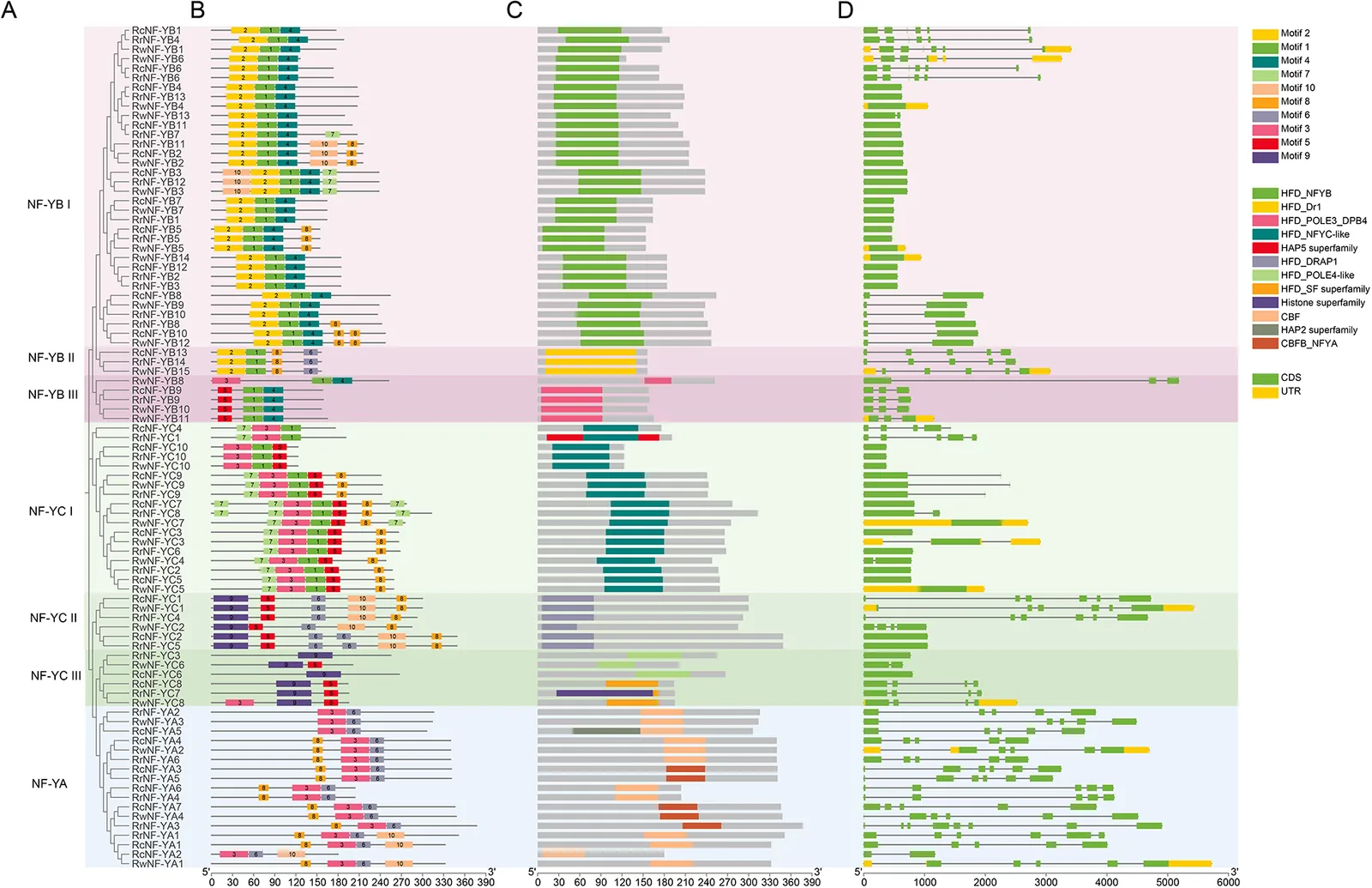
Phylogeny, conserved motifs, domains, and gene structures of *Rosa NF-Y*. **(A)** Phylogenetic tree of NF-Y proteins. **(B)** Identified motifs (motifs 1–10) in NF-Y proteins, with different colors representing distinct motifs. **(C)** Domain analysis of NF-Y proteins; different domains are shown as colored boxes. **(D)** Gene structures of *NF-Y* genes; green boxes indicate CDS, gray lines indicate introns, and yellow boxes indicate UTRs.

The composition and distribution of conserved motifs closely mirrored the domain analysis, displaying prominent subfamily- and subgroup-specific patterns (**Figures 4A, B**; **Supplementary Table S7**). In the NF-YA subfamily, a core binary motif (motif 3 + motif 6) was present in all members, with additional motifs (motif 8 and/or motif 10) flanking this core in a member-specific manner. Among NF-YB subgroups, subgroup I featured a characteristic triple motif (motif 2–motif 1–motif 4), whereas subgroups II and III displayed variant arrangements in which specific motifs were gained, lost, or repositioned. The NF-YC subgroups exhibited the most pronounced motif divergence: subgroup I consistently carried a core motif 3–motif 1 pair with variable N- and C-terminal extensions (motif 7 and/or motif 5), subgroup II displayed a fixed linear order (motif 9–motif 5–motif 6–motif 10–motif 8), and subgroup III had the simplest composition, with most members containing only a single motif 9 or a motif 9–motif 5 pair. Detailed motif compositions for each member are provided in **Supplementary Table S7**.

Regarding gene structure, the features among subfamilies and subgroups were less pronounced than those of domains and conserved motifs, with some overlap between *NF-YB* and *NF-YC* (**Figures 4A, D**). Overall, *NF-YB* subgroup I and *NF-YC* subgroup I mainly contained single-exon genes, whereas *NF-YB* subgroups II and III, as well as NF-YC subgroups II and III, predominantly consisted of multi-exon genes; notably, all members of *NF-YB* subgroups II and III were multi-exon genes. In the *NF-YA* subfamily, except for *RcNF-YA2* which contained only two exons, all other members had at least five exons. Integrating the domain, conserved motif, and gene structure analyses, the *Rosa NF-Y* family exhibited a pattern of conserved functional domains but variable gene structures during evolution: the retention of core functional domains and key motifs maintained the basic functions of each subfamily, whereas variations in gene structure (exon number) may have facilitated functional divergence among different subgroups.

### 3.5 Protein-protein interaction network analysis of NF-Y proteins

NF-Y proteins typically function by forming NF-YA/NF-YB/NF-YC heterotrimeric complexes to regulate plant development, stress responses, and various biological processes. To explore the interaction mechanisms of *Rosa* NF-Y proteins, protein-protein interaction (PPI) networks were predicted and constructed for the three *Rosa* species (**Figure 5**; **Supplementary Table S8**). The results showed that all NF-Y proteins, except for the absence of one RwNF-YC2 member in *R. wichuraiana*, participated in the PPI networks. In *R. chinensis*, 12 core members were identified (**Figure 5A**), including three NF-YA (RcNF-YA1, RcNF-YA5, and RcNF-YA7), three NF-YB (RcNF-YB2, RcNF-YB4, and RcNF-YB11), and six NF-YC (RcNF-YC3, RcNF-YC4, RcNF-YC5, RcNF-YC7, RcNF-YC9, and RcNF-YC10) proteins. In *R. rugosa*, 11 core members were identified (**Figure 5B**), including three NF-YA (RrNF-YA1, RrNF-YA2, and RrNF-YA3), three NF-YB (RrNF-YB7, RrNF-YB11, and RrNF-YB13), and five NF-YC (RrNF-YC2, RrNF-YC6, RrNF-YC8, RrNF-YC9, and RrNF-YC10) proteins. In *R. wichuraiana*, the number of core members decreased to nine (**Figure 5C**), including three NF-YA (RwNF-YA1, RwNF-YA3, and RwNF-YA4) and six NF-YC (RwNF-YC3, RwNF-YC4, RwNF-YC5, RwNF-YC7, RwNF-YC9, and RwNF-YC10) proteins, with no NF-YB member identified as core. Notably, these core interacting NF-Y proteins were phylogenetically close to each other on the tree (**Figure 5B**). Specifically, all core NF-YB members (RcNF-YB2, RcNF-YB4, RcNF-YB11, RrNF-YB7, RrNF-YB11, and RrNF-YB13) belonged to NF-YB subgroup I; all core NF-YC members were from NF-YC subgroup I, and only RrNF-YC1 in this subgroup was not a core protein. These findings suggest that NF-YC subgroup I is a key component of the NF-YA/NF-YB/NF-YC heterotrimer, and the assembly pattern of this heterotrimer is conserved across *Rosa* species.

**Figure 5:**
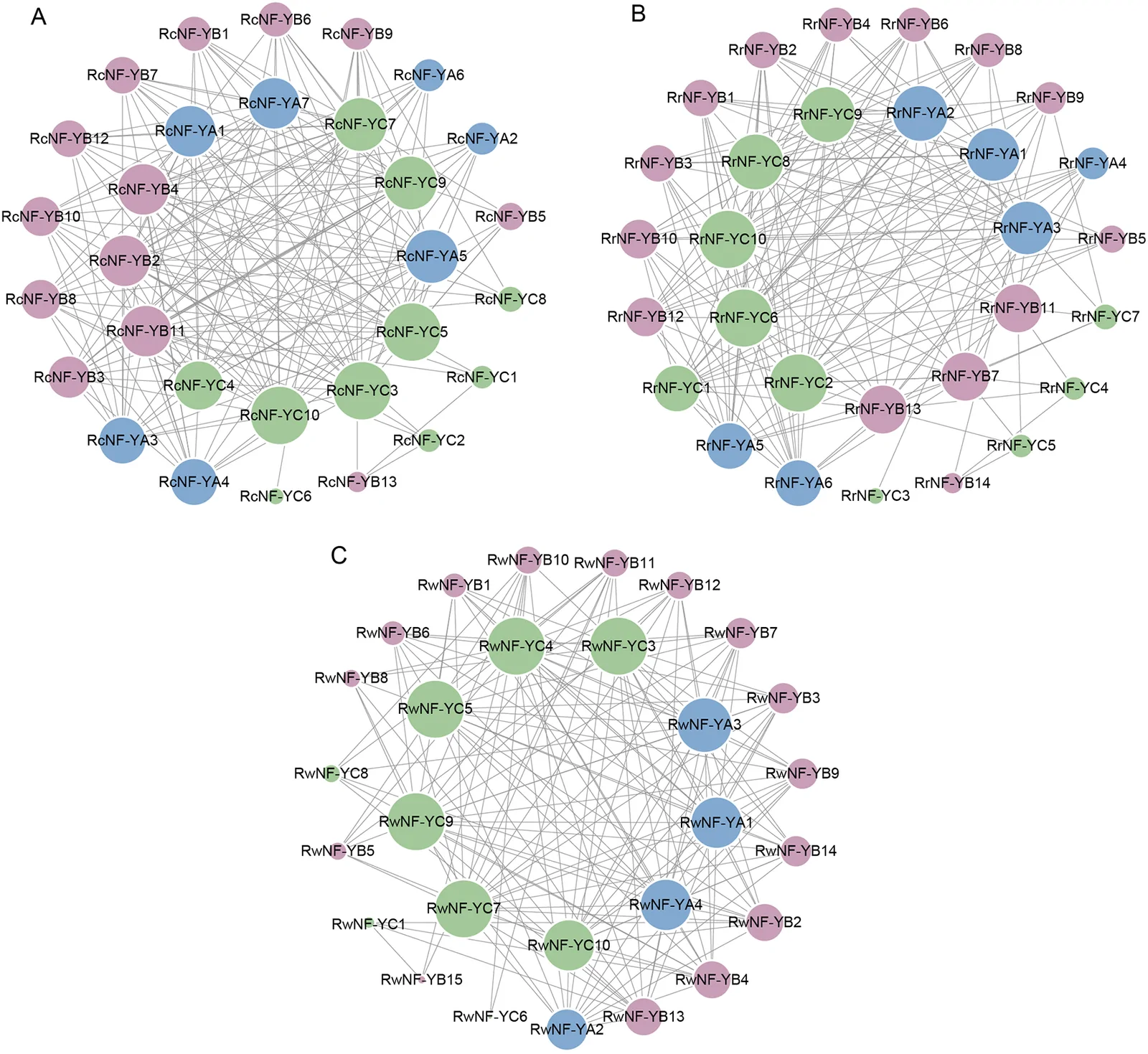
Protein-protein interaction networks of NF-Y proteins from *R. chinensis* **(A)**, *R. rugosa* **(B)**, and *R. wichuraiana* **(C)**. Different colors represent different subfamily members; circle size indicates the degree of interactions (number of connections); lines represent interactions between proteins.

### 3.6 Composition and distribution of cis-acting elements in *NF-Y* gene promoters

To investigate the potential transcriptional regulatory mechanisms of *Rosa NF-Y* genes, cis-acting element analysis was performed on the promoter regions of 89 *NF-Y* genes. A total of 50 elements were identified and classified into four categories, comprising 23, 10, 7, and 10 elements, respectively (**Figure 6**). Among light-responsive elements, Box4 and G-Box were the most prevalent, being present in at least one copy in over 80% of *NF-Y* gene promoters; some members contained up to five or six copies of Box4 or G-Box. Among phytohormone-responsive elements, the abscisic acid (ABA)-responsive ABRE element and the methyl jasmonate (MeJA)-responsive CGTCA-motif and TGACG-motif were predominant. ABRE elements were widely distributed, absent in only 10 gene promoters, which mainly belonged to *NF-YB* subgroup III, *NF-YC* subgroup II, and *NF-YC* subgroup III; in contrast, approximately 40% of members in *NF-YB* subgroup I and *NF-YC* subgroup I contained no fewer than four ABRE elements in their promoters. Growth- and development elements were relatively few and were scattered across individual gene promoters. Among stress-responsive elements, the anaerobic response-related ARE element was the most abundant, absent in only six gene promoters; 65 genes (73%) possessed two or more ARE elements, and 39 genes (43.8%) possessed three or more ARE elements.

**Figure 6:**
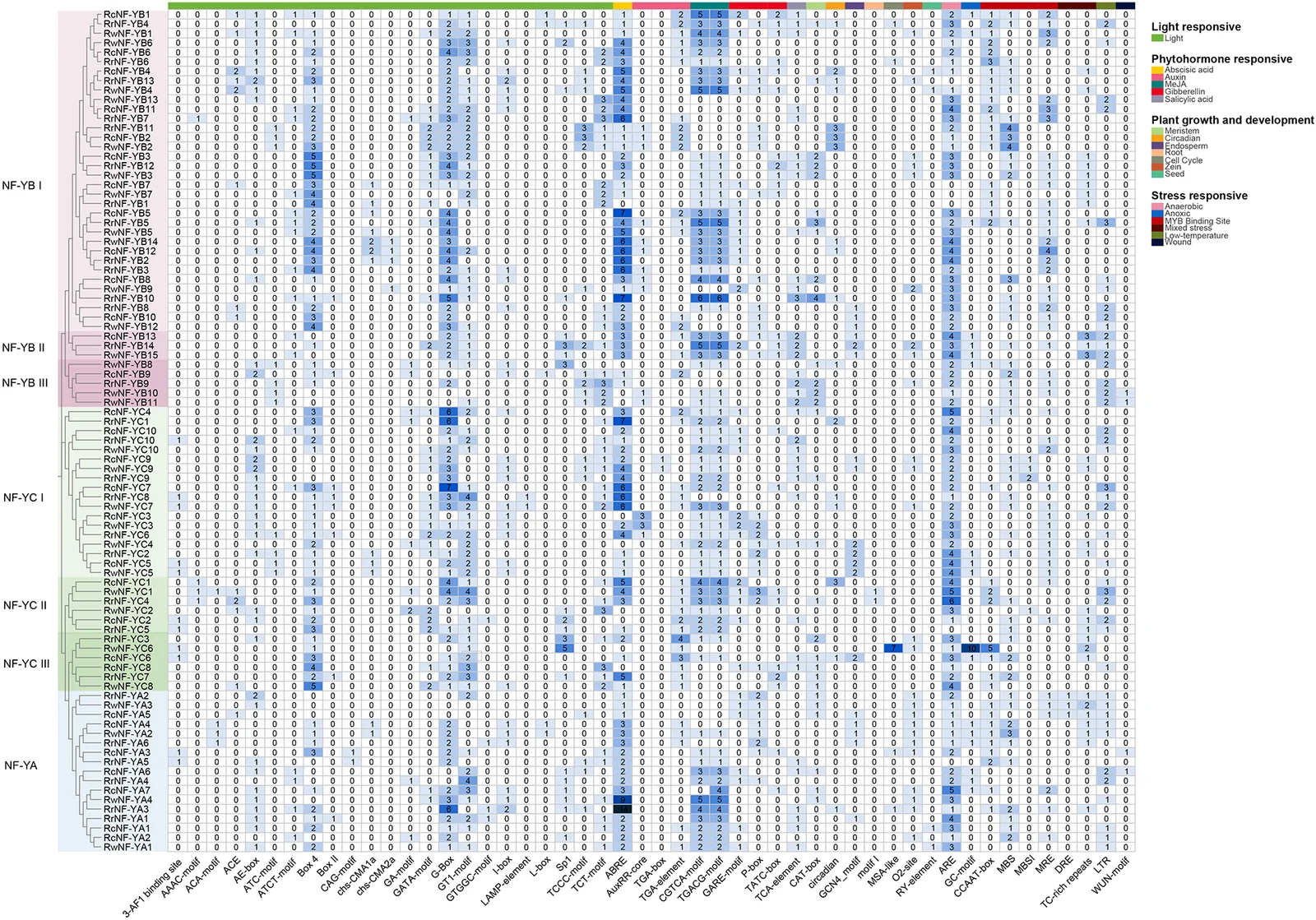
Heatmap of cis-acting element counts in the promoters of *Rosa NF-Y* genes. The left phylogenetic tree distinguishes subfamilies and subgroups with different color blocks; the upper color-coded bars represent different categories of cis-acting elements; heatmap color intensity indicates element counts, with darker colors representing higher counts.

Further mapping of cis-element distribution along the promoters revealed that, unlike the organized patterns of domains and conserved motifs (**Figure 4**), the composition and distribution of promoter elements did not exhibit obvious subfamily-specific or species-consistent patterns overall (**Supplementary Figure 2**). Nevertheless, within certain evolutionary branches, *Rosa* orthologous genes still showed relatively conserved element composition and distribution patterns. For example, in the *NF-YB* subgroup I, the branches *RcNF-YB2*/*RrNF-YB11*/*RwNF-YB2*, *RcNF-YB3*/*RrNF-YB12*/*RwNF-YB3*, and *RcNF-YB12*/*RrNF-YB2*/*RrNF-YB3*/*RwNF-YB14*; in *NF-YC* subgroup I, the branches *RcNF-YC3*/*RrNF-YC6*/*RwNF-YC3* and *RcNF-YC5*/*RrNF-YC2*/*RwNF-YC5*; and in the *NF-YA* subfamily, the branches *RcNF-YA5*/*RrNF-YA2*/*RwNF-YA3* and *RcNF-YA4*/*RrNF-YA6*/*RwNF-YA2* all exhibited branch-specific conserved element distribution patterns. Interestingly, the average number of cis-elements in the *NF-YB* and *NF-YC* subfamilies was higher than that in the *NF-YA* subfamily, particularly for light-responsive and phytohormone-responsive elements (**Supplementary Table S9**). Collectively, the promoters of *Rosa NF-Y* genes harbor diverse environment-responsive cis-acting elements, with a distribution that is branch-specific rather than strictly conserved across subfamilies. This suggests that different *NF-Y* members may play differentiated regulatory roles in light, hormone, and stress signaling pathways, a possibility that warrants experimental validation.

### 3.7 Expression patterns of *NF-Y* genes in different tissues

To systematically dissect the tissue expression characteristics of *Rosa NF-Y* genes, publicly available transcriptomic data were analyzed for six tissues of the *R. chinensis* cultivar ‘Old Blush’, four tissues of *R. wichuraiana*, and five tissues of *R. rugosa*. Heatmap visualization revealed expression pattern divergence both within and between *NF-Y* subfamilies, whereas evolutionarily close orthologs among species tended to exhibit relatively similar expression profiles (**Figure 7**). For instance, high-expression branches included *RcNF-YB1/RrNF-YB4/RwNF-YB1*, *RcNF-YB6/RrNF-YB6/RwNF-YB6*, *RcNF-YB4/RcNF-YB13/RwNF-YB4*, *RcNF-YB13/RrNF-YB14/RwNF-YB15*, and *RcNF-YC9/RrNF-YC9/RwNF-YC9*, all of which displayed moderate to high expression levels in all examined tissues. Conversely, low-expression branches such as *RcNF-YB8/RrNF-YB10/RwNF-YB9*, *RcNF-YB10/RrNF-YB8/RwNF-YB12*, and *RcNF-YC10/RrNF-YC10/RwNF-YC10* exhibited extremely low expression across all tissues. Despite these conserved expression trends, some orthologous branches displayed divergent expression patterns among species. Taking the branch *RcNF-YB9/RrNF-YB9/RwNF-YB8/RwNF-YB10/RwNF-YB11* as an example: *RcNF-YB9* showed significantly higher expression in the stem of *R. chinensis* than in other tissues; *RrNF-YB9* exhibited low expression in rose petals but moderate levels in other tissues; *RwNF-YB8* showed very low expression in all four tissues of *R. wichuraiana*; *RwNF-YB10* was highly expressed in buds; and *RwNF-YB11* displayed moderate and consistent expression across tissues. Another example is the branch *RcNF-YC5/RrNF-YC2/RwNF-YC4/RwNF-YC5*, where all members except *RwNF-YC4* showed the highest expression in roots of their respective species. In the branches *RcNF-YC1/RrNF-YC4/RwNF-YC1*, *RcNF-YC2/RrNF-YC5/RwNF-YC2*, and *RcNF-YA7/RrNF-YA3/RwNF-YA4*, the rose counterparts were almost undetectable in all tissues, whereas the *R. chinensis* and *R. wichuraiana* counterparts exhibited moderate to high stable expression across all tissues. Taken together, these expression profiles reveal both conservation and divergence in the tissue-specific deployment of *NF-Y* genes across *Rosa* species, with orthologous branches generally maintaining similar expression patterns while species-specific shifts contribute to phenotypic diversification.

**Figure 7:**
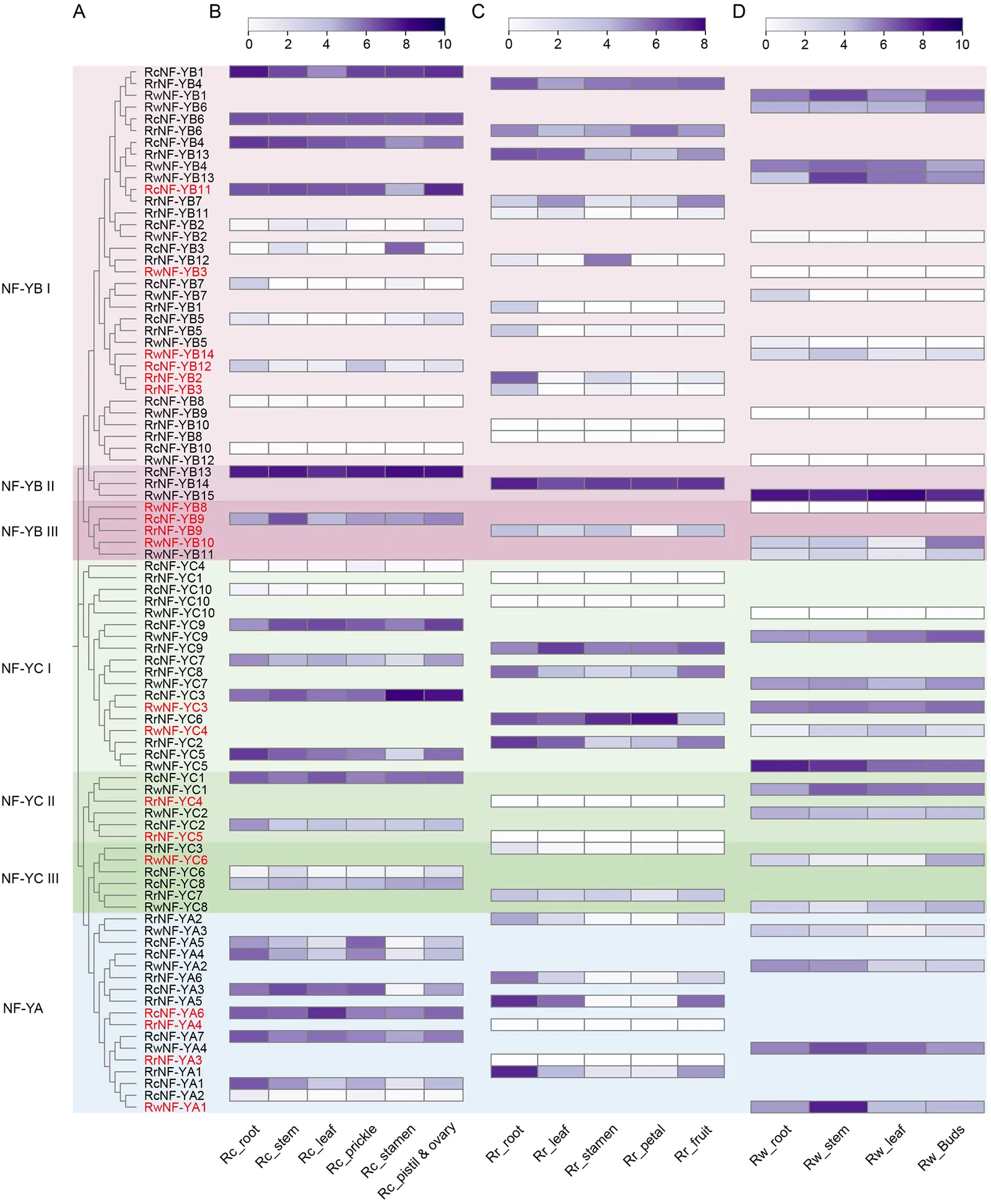
Expression profiles of *Rosa NF-Y* genes in different tissues. **(A)** Phylogenetic tree of *Rosa* NF-Y proteins. **(B)** Heatmap of *RcNF-Y* gene expression in six tissues of *R. chinensis* ‘Old Blush’. **(C)** Heatmap of *RrNF-Y* gene expression in five tissues of *R. rugosa*. **(D)** Heatmap of *RwNF-Y* gene expression in four tissues of *R. wichuraiana*. Expression levels are represented as log_2_(FPKM) values, with colors from light to dark corresponding to low to high expression levels.

### 3.8 Differential responses of *RcNF-Y* and *RrNF-Y* genes to early heat stress

Heat stress is a major abiotic stress factor affecting the growth and development of *Rosa* species. Previous studies have shown that short-term high-temperature treatment reduces photosynthetic efficiency and increases membrane lipid peroxidation in the *R. chinensis* cultivar ‘Slater’s crimson China’ and the *R. rugosa* cultivar ‘BaoWhite’, indicating that heat stress significantly impacts the physiological metabolism of these two species (Li ZQ. et al., 2019; Ding et al., 2024). To explore the potential roles of *NF-Y* genes in early heat stress response, expression changes of *NF-Y* genes in these two species after short-term high-temperature treatment (2 hours) were analyzed (**Figure 8**). Differential expression analysis revealed that five *NF-Y* genes in *R. chinensis* responded to heat stress, including three up-regulated genes (*RcNF-YB1*, *RcNF-YB4* and *RcNF-YC2*) and two down-regulated genes (*RcNF-YA4* and *RcNF-YA6*) (**Figure 8A**). In *R. rugosa*, eight *NF-Y* genes responded to heat stress, including four up-regulated genes (*RrNF-YA2*, *RrNF-YB4*, *RrNF-YB14* and *RrNF-YC9*) and four down-regulated genes (*RrNF-YA1*, *RrNF-YA4*, *RrNF-YA6* and *RrNF-YC7*) (**Figure 8B**). Notably, some orthologous gene pairs between *R. chinensis* and *R. rugosa* exhibited different response patterns under heat stress. For example, *RrNF-YC9* was significantly up-regulated in *R. rugosa* after heat stress, whereas its ortholog *RcNF-YC9* showed non-significant or even slight down-regulation in *R. chinensis*; *RrNF-YA1* was significantly down-regulated in *R. rugosa*, whereas its ortholog *RcNF-YA1* showed non-significant or slight up-regulation in *R. chinensis*; *RrNF-YA2* was significantly up-regulated in *R. rugosa*, whereas its ortholog *RcNF-YA5* was filtered out due to extremely low expression levels. Collectively, *NF-YA* subfamily members contributed the largest number of heat-responsive genes across the two species (two in *R. chinensis* and four in *R. rugosa*), but the heat stress response patterns of *NF-Y* genes differed markedly between the two species, which may be associated with their distinct heat adaptation mechanisms.

**Figure 8:**
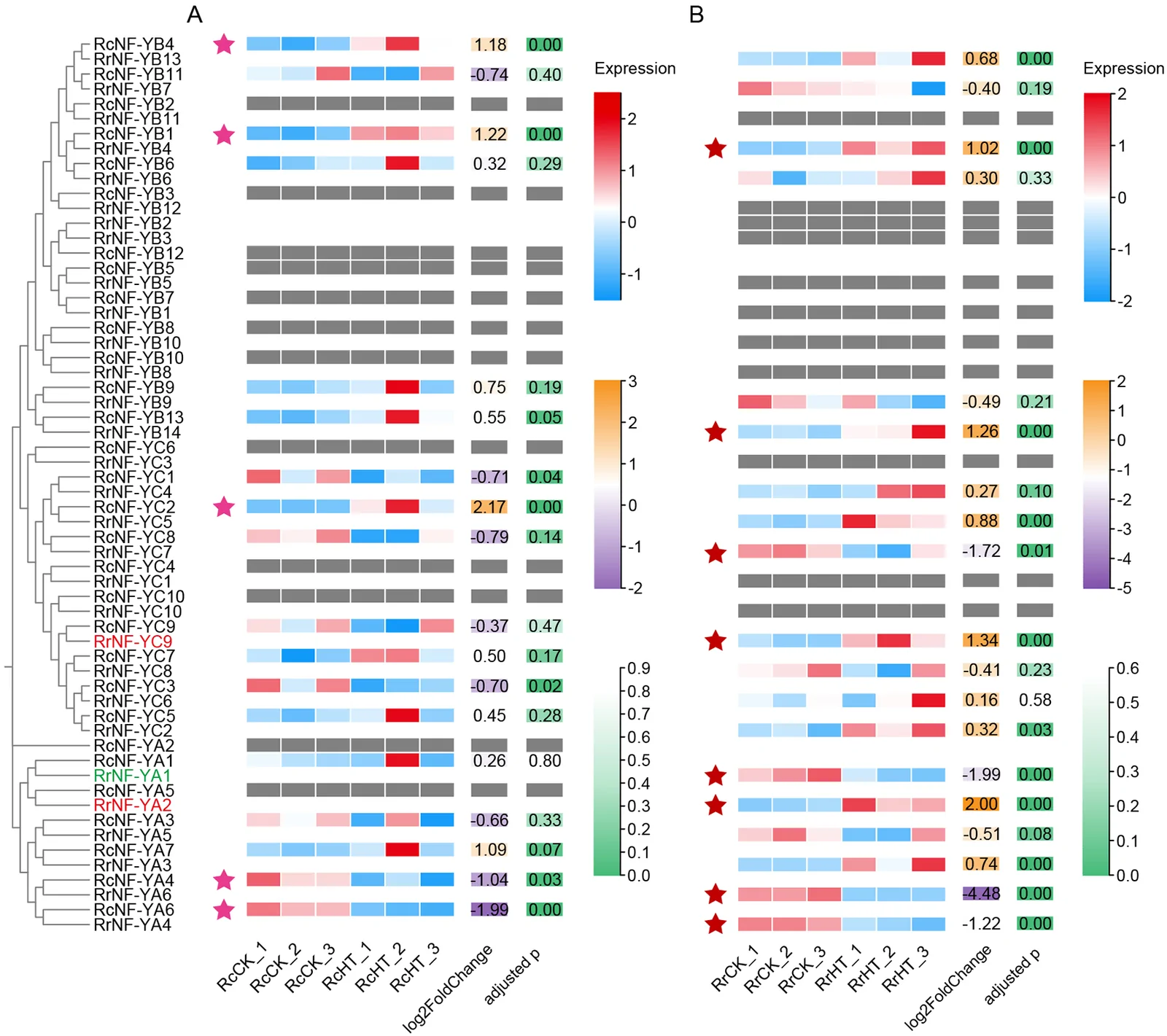
Response patterns of *R. chinensis* **(A)** and *R. rugosa* **(B)** *NF-Y* genes to heat stress. Red and blue blocks indicate normalized gene expression levels; orange-purple blocks indicate log_2_FoldChange values; green blocks indicate adjusted p for differential expression significance; gray blocks indicate genes filtered out by DESeq2 due to low expression; red and pink stars indicate *NF-Y* genes significantly differentially expressed under heat stress (|log_2_FoldChange| ≥ 1 and adjusted p < 0.05).

### 3.9 Involvement of *RcNF-Y* genes in the regulation of secondary metabolite biosynthesis

Rose is rich in various secondary metabolites, including flavonoids and terpenoids. Previous studies have shown that *NF-Y* can regulate flavonoid biosynthesis in tomato (Wang et al., 2021); however, whether *NF-Y* participates in secondary metabolic regulation in rose remains unclear. To address this question, transcriptome sequencing was first performed on six roses (BLLW, PLII, HBK, HLL, YYH, JSQ). Compared with white-colored cultivars, a large number of differentially expressed genes (DEGs) were identified in both red- and yellow-colored cultivars. Specifically, 4,420 and 2,554 genes were up-regulated, and 2,863 and 2,225 genes were down-regulated in red- and yellow-colored cultivars, respectively (**Supplementary Figures 3A, B**). WGCNA based on these DEGs partitioned the genes into 15 co-expression modules (**Supplementary Figure 4**). Nine *RcNF-Y* genes were distributed across five of these modules (**Supplementary Table S10**), and module-trait correlation analysis associated each module with a specific cultivar (correlation coefficients and adjusted p-values are shown in **Figure 9A**): *RcNF-YC2* in the blue module (correlated with PLII); *RcNF-YB13* in the brown module (correlated with BLLW); *RcNF-YB1* in the green module (correlated with HLL); *RcNF-YA3* and *RcNF-YB6* in the turquoise module (correlated with HBK); and *RcNF-YA1*, *RcNF-YA4*, *RcNF-YA5*, and *RcNF-YB12* in the yellow module (correlated with YYH). To further clarify the metabolic pathways enriched in these modules, KEGG enrichment analysis was performed on each of the five modules. The results showed that all modules were enriched to varying extents in secondary metabolic pathways, including phenylpropanoid biosynthesis, flavonoid biosynthesis, terpenoid backbone biosynthesis, monoterpenoid biosynthesis, and tropane, piperidine, and pyridine alkaloid biosynthesis (**Figure 9B**; **Supplementary Table S11**), suggesting that *RcNF-Y* genes may participate in the regulation of flavonoids, terpenoids, and alkaloids.

**Figure 9:**
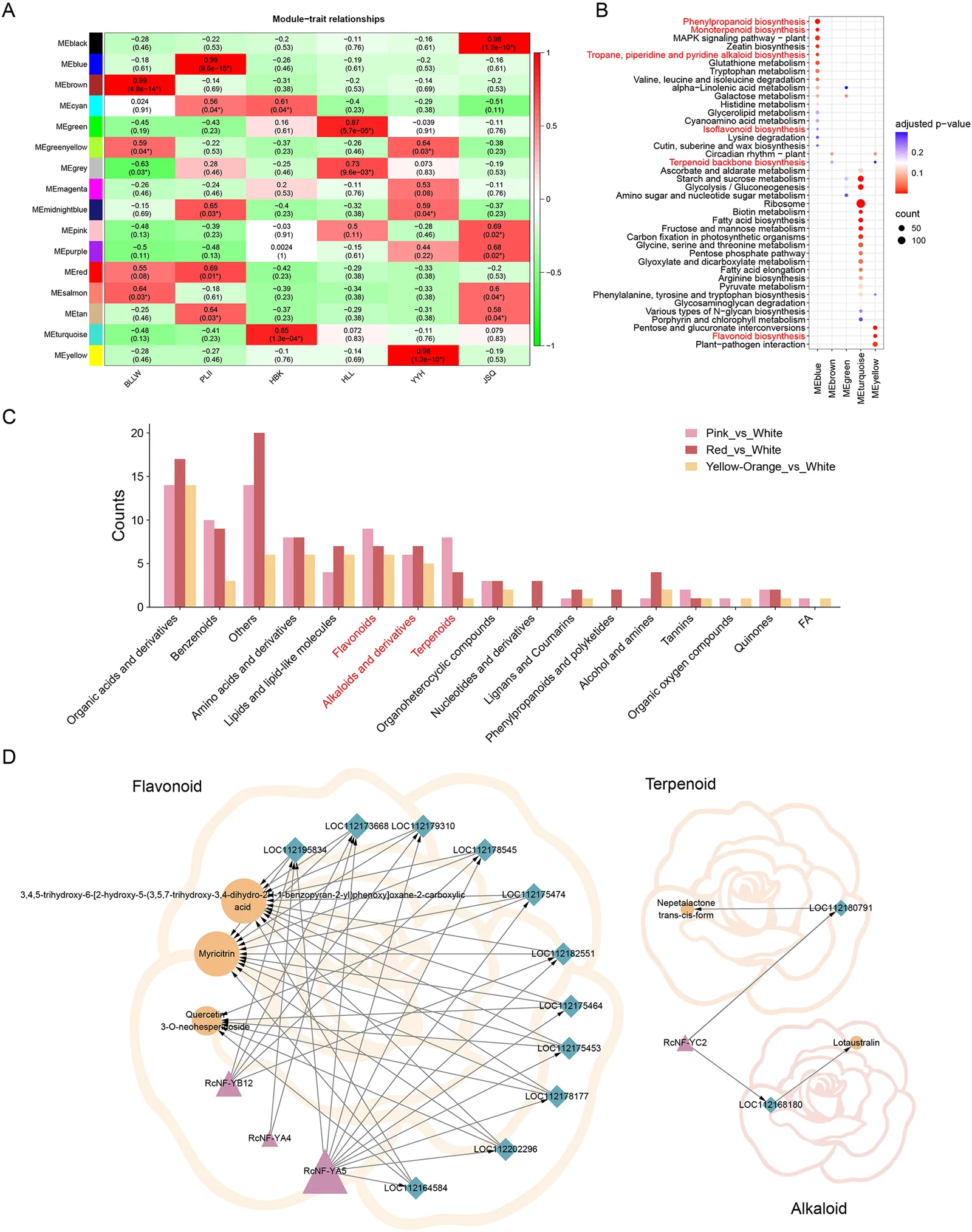
WGCNA and untargeted metabolomics results of different roses. **(A)** Heatmap of module-trait relationships showing correlations between co-expression module eigengenes and cultivar-group traits, with correlation coefficients and adjusted p-values. **(B)** KEGG pathway enrichment results for modules containing *RcNF-Y* genes. **(C)** Classification of differentially accumulated metabolites between different color series of roses. **(D)** Correlation network of *RcNF-Y* genes, key genes involved in the biosynthesis of the three metabolite classes, and representative metabolites.

To further validate this hypothesis, an additional red-colored cultivar (JNBBL), two pink-colored cultivars (XZYY, YYF), and one orange-colored cultivar (ZZ) were included, and untargeted metabolomics analysis was performed on all materials. Unsupervised principal component analysis (PCA) of the metabolite profiles clearly separated the different color groups, confirming genuine metabolic divergence among them (**Supplementary Figure 5**). Compared with white-colored cultivars, numerous differentially accumulated metabolites (DAMs) were identified in red-, pink-, and yellow-/orange-colored cultivars, with flavonoids, terpenoids, and alkaloids being among the top categories (**Figure 9C**; **Supplementary Table S12**-**S14**). Heatmap visualization further revealed variations in the contents of these three metabolite classes among different roses (**Supplementary Figure 6**). Based on the transcriptomic and metabolomic data, a correlation network was constructed among *RcNF-Y* genes, genes involved in the biosynthesis of the three metabolite classes, and the metabolites themselves (**Figure 9D**; **Supplementary Table S15**). The flavonoid regulatory network exhibited high complexity, with three *NF-Y* members (*RcNF-YB12*, *RcNF-YA4*, and *RcNF-YA5*) tightly associated with multiple structural genes and flavonoid metabolites, including myricitrin and quercetin-3-O-neohesperidoside. Notably, *RcNF-YA5* served as a hub with abundant connection edges, suggesting a central role in modulating flavonoid biosynthesis. In contrast, the terpenoid and alkaloid pathways displayed relatively simple regulatory modules: *RcNF-YC2* was linked to the structural gene *LOC112168180*, which further correlated with the alkaloid metabolite lotaustralin, while the terpenoid metabolite nepetalactone trans-cis-form was associated only with *LOC112180791*. Collectively, these network patterns imply that distinct *RcNF-Y* members may exert divergent regulatory functions in the biosynthesis of flavonoids, terpenoids, and alkaloids.

## 4 Discussion

### 4.1 Evolutionary characteristics and structural divergence of *Rosa NF-Y* genes

In this study, we systematically identified and compared the NF-Y transcription factor families in three representative *Rosa* species—*R. chinensis*, *R. rugosa*, and *R. wichuraiana*—at the genome-wide level. A total of 89 *NF-Y* genes were identified, with similar numbers in the three species (30, 30, and 29, respectively), indicating that this family has remained relatively conserved during *Rosa* evolution. Compared with *A. thaliana* (36 genes) (Siefers et al., 2009) and *O. sativa* (40 genes) (Manimaran et al., 2017), the total number of *NF-Y* genes in *Rosa* is slightly lower, primarily due to reductions in the *NF-YA* and *NF-YC* subfamilies. This numerical difference may be attributed to lineage-specific gene loss or species-specific expansion, similar to observations in peach (24 genes) (Li M. et al., 2019) and grapevine (32 genes) (Ren et al., 2016).

Synteny analysis revealed that segmental duplication is the primary mechanism driving *NF-Y* gene expansion in *Rosa*, consistent with findings in poplar (Liu et al., 2021) and peach (Li M. et al., 2019). The Ka/Ks ratios of all duplicated gene pairs, except those for which Ks could not be calculated, were substantially less than 1, indicating that purifying selection has dominated the evolution of *Rosa NF-Y* genes to maintain the stability of core functions—a finding consistent with observations in kiwifruit (Zhang et al., 2026) and soybean (Quach et al., 2015).

Notably, the NF-YB and NF-YC subfamilies in *Rosa* could be further divided into three subgroups with marked structural differences. In particular, NF-YB subgroup II (containing the HFD_Dr1 domain, homologous to the NC2β/Dr1 subunit) and NF-YC subgroup II (containing the HFD_DRAP1 domain, homologous to the NC2α/DRAP1 subunit) harbor histone-fold domains characteristic of the general transcriptional co-repressor NC2 rather than of canonical NF-Y subunits (Petroni et al., 2012). In humans and yeast, NC2 functions as a global transcriptional repressor by binding to the TATA-binding protein (TBP) to inhibit transcription initiation (Kamada et al., 2001); notably, the NC2 subunits are biochemically incompatible with NF-Y subunits and do not form complexes with them (Zemzoumi et al., 1999). We therefore speculate that these subgroups in *Rosa* may represent functionally diverged NF-Y-like proteins that, instead of participating in canonical CCAAT-box binding, may exert transcriptional repression or unique regulatory functions. Notably, homologous proteins in Arabidopsis and rice that belong to the same evolutionary lineage as these divergent NF-Y-like proteins of *Rosa* have been described as members of the *NF-Y* family (**Figure 2A**). Protein sequence alignment further reflected the sequence similarity between these divergent NF-Y-like proteins of *Rosa* and the aforementioned known NF-Y family proteins, thereby confirming the reliability of our identification results (**Supplementary Figures 7**-**10**). Such subgroup classification has rarely been reported in previous plant *NF-Y* studies and reveals a distinctive structural diversification feature of the *NF-Y* family in *Rosa*.

### 4.2 Conservation of protein-protein interaction networks and core components

PPI network prediction showed that the core interacting members common to all three species were exclusively from NF-YC subgroup I (e.g., RcNF-YC3, RrNF-YC6) and NF-YB subgroup I (e.g., RcNF-YB2, RrNF-YB7), which is highly consistent with the classical model in which NF-YB and NF-YC subunits must first form heterodimers (Hackenberg et al., 2012). In addition, core NF-YA members (e.g., RcNF-YA1, RrNF-YA1) also participated extensively in this network. Notably, almost all NF-YC members predicted to be involved in core interactions belonged to NF-YC subgroup I, suggesting that this subgroup may serve as the key scaffold for the formation of functional NF-YA/NF-YB/NF-YC heterotrimers. The presence of the typical HFD_NFYC-like domain in all members of this subgroup further supports their predicted central role as an “interaction platform”.

Meanwhile, some non-core interacting NF-Y members, such as RcNF-YA4 and RcNF-YB12, although not meeting the degree threshold for core status, still exhibited interactions with other proteins in the network, particularly with the core protein RcNF-YC9 (**Supplementary Table S7**). Intriguingly, in our WGCNA analysis, these two genes were co-localized in the yellow module and showed strong correlations with flavonoid metabolites. These observations suggest that canonical NF-Y complexes assembled from non-core subunits may be involved in the fine-tuned regulation of specific metabolic pathways.

### 4.3 Functional implications of promoter cis-elements and expression divergence

Promoter analysis revealed that *Rosa NF-Y* genes are enriched in numerous hormone-responsive elements, including ABRE and CGTCA-motif. This finding aligns with previous observations in this study and in other plants (Li et al., 2008; Zhang et al., 2026) that *NF-Y* genes are induced by signals such as ABA and methyl jasmonate, suggesting that *Rosa NF-Y* may potentially participate in hormone-mediated stress adaptation, although these promoter cis-element predictions require functional validation. Notably, ABRE elements are highly enriched in *NF-YB* subgroup I and *NF-YC* subgroup I, which contain most of the core interacting members, implying that these “core” NF-Y proteins may serve as important downstream regulators of ABA signaling pathways. In Arabidopsis, ABF3/4 transcription factors form a complex with NF-YC3/4/9 to co-activate *SOC1* expression, thereby mediating early flowering under drought stress (Hwang et al., 2019); this mechanism depends on ABRE elements. Hence, ABRE-rich *NF-Y* genes in *Rosa* may similarly participate in ABA-dependent stress response pathways.

Tissue expression profiling further confirmed that *Rosa* orthologs with high sequence similarity (e.g., the *RcNF-YB1*/*RrNF-YB4*/*RwNF-YB1* branch) tend to exhibit conserved expression patterns, whereas members of certain branches (e.g., *NF-YB* subgroup III) have undergone marked expression divergence among species. This expression-level divergence may serve as a molecular basis for species-specific traits, such as differences in floral scent, flower morphology, and stress tolerance between *R. rugosa* and *R. chinensis*. Notably, *RcNF-YC2* showed moderate expression levels in multiple tissues of *R. chinensis*, whereas its ortholog *RrNF-YC5* in *R. rugosa* exhibited a different expression pattern, which may be associated with inter-species differences in secondary metabolite accumulation and stress resistance.

### 4.4 Potential roles of NF-Y: heat stress response and secondary metabolic regulation

#### 4.4.1 Species-specific responses of *NF-Y* genes to heat stress in *Rosa*

In this study, we found that several *NF-Y* genes, particularly those in the *NF-YA* subfamily, responded to short-term high-temperature treatment in both *R. chinensis* and *R. rugosa*. However, the response patterns differed significantly between the two species, with some orthologous pairs showing opposing regulation (e.g., *RcNF-YC9* vs. *RrNF-YC9*; *RcNF-YA1* vs. *RrNF-YA1*). This observation is partially consistent with the well-documented involvement of *NF-YA* members in heat stress responses in Arabidopsis, tomato and Ginkgo (Rao et al., 2022; Wang et al., 2023), but also highlights that the heat-responsive mechanisms of *NF-Y* in *Rosa* may be species-specific. The differential responses could be attributed to variations in promoter cis-element composition or upstream signaling pathways between the two species, which may reflect their different ecological adaptations—*R. rugosa* is known for higher stress tolerance compared to *R. chinensis* (Chen et al., 2021). Future functional validation, such as overexpression or CRISPR/Cas9 knockout of candidate heat-responsive *NF-Y* genes (e.g., *RcNF-YA5*, *RrNF-YA2*), is needed to confirm their roles in thermotolerance. It should also be noted that the two heat-stress datasets were obtained from independent public studies, which could contribute to the observed inter-species differences. Overall, this analysis provides the first evidence for the involvement of *NF-Y* genes in heat stress response in *Rosa* and offers candidate targets for breeding heat-tolerant rose cultivars.

#### 4.4.2 Potential roles of *RcNF-Y* genes in the regulation of flavonoid metabolism and flower color

In a separate line of investigation, our WGCNA and metabolomics analyses revealed that multiple *RcNF-Y* genes, especially *RcNF-YC2*, are closely associated with the accumulation of flavonoids, terpenoids, and alkaloids in different rose cultivars. This finding is consistent with the emerging role of *NF-Y* in secondary metabolism, as previously reported in tomato where NF-YB8/NF-YC1/NF-YA1/9 regulates *CHS1* expression and flavonoid accumulation (Wang et al., 2021), while *NF-YA10* negatively regulates AsA biosynthesis (Chen et al., 2020). However, our study extends these observations to a woody ornamental plant and links NF-Y to a broader range of secondary metabolites, also including flavonoids (the principal pigments of flower color (Tanaka et al., 2008)). Our results showed that *RcNF-YA4*, *RcNF-YA5*, and *RcNF-YB12* are all involved in flavonoid metabolism, particularly with respect to Myricitrin (myricetin-3-rhamnoside). This flavonol can act as a “copigment” to undergo copigmentation with anthocyanins (Gómez-Míguez et al., 2006), and can also serve as a “fading factor”, causing a hypochromic effect during anthocyanin coloration (Bao et al., 2020). Interestingly, all three genes were located in the yellow module in the WGCNA results, which was closely associated with the red rose cultivar YYH, and this module also contained *RcNF-YA1*. Notably, in the PPI network, RcNF-YA1/4/5 were all predicted to interact with RcNF-YB3 and RcNF-YC9, while RcNF-YB12 was predicted to interact with RcNF-YA1/4/5 and RcNF-YC9, suggesting the possible existence of a complex comprising RcNF-YA1/4/5, RcNF-YB3/9, and RcNF-YC9 that finely regulates flavonoid metabolism in roses. However, this hypothesis requires experimental validation through yeast two-hybrid (Y2H) and bimolecular fluorescence complementation (BiFC) assays, as well as electrophoretic mobility shift assays (EMSA) and dual-luciferase reporter assays with a reconstituted NF-YA/NF-YB/NF-YC heterotrimer to confirm direct binding to the CCAAT elements of key biosynthetic gene promoters.

#### 4.4.3 A potential link between heat stress and terpenoid/alkaloid metabolism

Although we investigated heat stress responses and secondary metabolic regulation as separate functional dimensions in this study, accumulating evidence suggests that these processes may be mechanistically linked. High temperatures are known to suppress anthocyanin accumulation and modulate volatile terpenoid emission in rose petals (Dela et al., 2003; Zhang et al., 2025). Notably, *RcNF-YC2*, an NF-Y candidate, was identified in both contexts: it responded to heat stress (**Figure 8A**) and showed correlation with terpenoid (major determinants of floral fragrance and also play roles in plant thermotolerance (Mostafa et al., 2022; Nagalingam et al., 2023; Zuo et al., 2025)) and alkaloid (associated with plant defense (Pérez-Mesa and Roda, 2025)) metabolites in our WGCNA-metabolomics analysis (**Figure 9D**). This dual association raises the intriguing possibility that *RcNF-YC2* may serve as a molecular hub coordinating stress adaptation and metabolic output. Under heat stress, RcNF-YC2 and its interacting partners might be recruited to distinct target promoters, activating heat-protective genes while simultaneously modulating terpenoid and alkaloid biosynthetic genes, thereby rebalancing resource allocation between defense and secondary metabolism. Although our current data do not directly demonstrate this connection, future functional studies should prioritize *RcNF-YC2*. Interestingly, in our PPI network, RcNF-YC2 was predicted to interact only with several RcNF-YB members, with no RcNF-YA partner identified. To address this, we propose the following experimental strategies: (1) validate the physical interactions between RcNF-YC2 and its putative NF-YB/NF-YA partners via Y2H and yeast library screening, thereby reconstituting complete NF-YA/YB/YC or other TF/YB/YC heterotrimers; (2) virus-induced gene silencing (VIGS) or CRISPR/Cas9-mediated knockout of *RcNF-YC2* in rose petals, followed by heat treatment and metabolomic profiling, to directly assess its potential dual roles in thermotolerance and terpenoid accumulation; (3) dual-luciferase reporter and EMSA assays using reconstituted heterotrimers to determine whether the complex directly binds to CCAAT boxes or other cis-elements in the promoters of key terpenoid biosynthetic genes (e.g., members of the terpenoid synthase family). Parallel investigation of species-specific heat-responsive orthologs (e.g., *RcNF-YA5* vs. *RrNF-YA2*) via heterologous expression in Arabidopsis or tobacco will further clarify the evolutionary divergence of NF-Y-mediated thermotolerance in *Rosa*, and these efforts will ultimately illuminate how the NF-Y regulatory network coordinates growth, stress adaptation, and metabolic diversification in woody ornamental plants.

## 5 Conclusions

This study presents the first systematic comparative analysis of the *NF-Y* gene family across three *Rosa* species, revealing lineage-specific diversification driven by segmental duplication and purifying selection. A key finding is the refined subgroup classification of the NF-YB and NF-YC subfamilies, which uncovers putative NF-Y-like members harboring NC2/DPB-related histone-fold domains that may function as transcriptional repressors rather than classical CCAAT-box activators. Through integration of transcriptomics and metabolomics, we associate *RcNF-YA4*, *RcNF-YA5*, and *RcNF-YB12* with flavonoid accumulation relevant to flower pigmentation, and identify *RcNF-YC2* as a candidate potentially linking *NF-Y* regulation to terpenoid and alkaloid accumulation—traits of direct relevance to rose fragrance, defense, and stress tolerance—and characterize species-divergent *NF-Y* responses to early heat stress. These findings provide a rich resource of candidate genes for functional dissection and offer a framework for understanding how *NF-Y*-mediated regulatory networks contribute to the remarkable phenotypic diversity of *Rosa* species.

## Data Availability

The RNA-seq data generated in this study for the six roses have been deposited in the NCBI Sequence Read Archive (SRA) database under accession number PRJNA1498212 (http://www.ncbi.nlm.nih.gov/bioproject/1498212). The untargeted metabolomics data have been deposited in the OMIX repository, National Genomics Data Center (NGDC), China National Center for Bioinformation, under accession number OMIX021011 (https://ngdc.cncb.ac.cn/omix/release/OMIX021011). Publicly available datasets used in this study are available from NCBI SRA under accession numbers: PRJNA546486 for six tissues of *R. chinensis* ‘Old Blush’, PRJNA436590 for four tissues of *R. wichuraiana*.
