# Supplementary material for: The NF-Y gene family in Rosa: evolutionary divergence across three species and potential roles in heat tolerance and secondary metabolism
Source: Front Plant Sci. 2026 Oct 8;17:1944775. doi: 10.3389/fpls.2026.1944775 (PMC13687345; doi:10.3389/fpls.2026.1944775)
Supplement: Supplementary file 1 [file DataSheet1.docx]

**Figure S1. Boxplots of intra‑ and inter‑species sequence similarity distributions of NF‑Y proteins among three *Rosa* species.** Left panel: intra‑species comparisons within each species. Right panel: inter‑species orthologous comparisons between species pairs. The y‑axis indicates sequence similarity (%). Red words represent comparisons within the same subfamily (intra‑subfamily); black words represent comparisons between NF‑YA and NF‑YB or NF‑YA and NF‑YC; blue words represent comparisons between NF‑YB and NF‑YC.

**Figure S2. Phylogenetic tree and promoter cis‑acting element distribution of *NF‑Y* genes in three *Rosa* species.** The phylogenetic tree on the left classifies *NF‑Y* genes into *NF‑YA*, *NF‑YB* (subgroups I/II/III), and *NF‑YC* (subgroups I/II/III). On the right, the distribution of cis‑acting elements in the 2 kb promoter regions upstream of each gene is shown. Colored vertical lines represent different functional cis‑acting elements. The horizontal axis indicates the promoter length (bp).

**Figure S3. Volcano plots of differentially expressed genes (DEGs) in red‑colored cultivars (A) and yellow‑colored cultivars (B) compared with white‑colored cultivars.** Red dots represent up‑regulated genes, blue dots represent down‑regulated genes, and gray dots indicate non‑significant changes.

**Figure S4. Results of weighted gene co‑expression network analysis (WGCNA).** (A) Soft‑thresholding power selection. A power of 14 was chosen to construct a scale‑free co‑expression network. (B) Sample clustering dendrogram showing no outlier samples. (C) Clustering dendrogram of module eigengenes and heatmap of module correlations. Different colors represent distinct co‑expression modules. The color intensity in the heatmap reflects the degree of correlation between module expression patterns.

**Figure S5. Principal component analysis (PCA) score plot of metabolite profiles from four color groups of *Rosa* accessions (White, Yellow‑Orange, Pink, and Red).** PC1 and PC2 explained 27.8% and 13.12% of the total variance, respectively, showing a clear separation among the four groups.

**Figure S6. Heatmaps showing normalized abundances of (A) Flavonoids, (B) Terpenoids, and (C) Alkaloids.** Rows represent individual metabolites (with left clustering dendrograms), and columns represent different samples. Sample abbreviations: BLLW (Bolero), PLII (Pope John Paul II), HBK (Black Baccara), HLL (Huo Linglong), YYH (Slater’s Crimson China), JNBBL (Memorial Barbara), XZYY (Xizi Yanyu), YYF (Old Blush), JSQ (Canary), ZZ (Masora). The color bar at the top distinguishes four sample groups. Red and blue colors indicate high and low standardized accumulation levels of metabolites, respectively.

**Figure S7. Amino acid sequence alignment of NF‑YB subgroup II proteins from *Rosa* species with orthologous from *Arabidopsis thaliana* and *Oryza sativa*.** The typical domains (HFD_Dr1 and CBFD_NFYB_HMF) are indicated by black solid lines.

**Figure S8. Amino acid sequence alignment of NF‑YB subgroup III proteins from *Rosa* species with orthologous from *Arabidopsis thaliana* and *Oryza sativa*.** The typical domains (HFD_POLE3_DPB4 and CBFD_NFYB_HMF) are indicated by black solid lines.

**Figure S9. Amino acid sequence alignment of NF‑YC subgroup II proteins from *Rosa* species with orthologous from *Arabidopsis thaliana* and *Oryza sativa*.** The typical domains (HFD_DRAP1 and CBFD_NFYB_HMF) are indicated by black solid lines.

**Figure S10. Amino acid sequence alignment of NF‑YC subgroup III proteins from *Rosa* species with orthologous from *Arabidopsis thaliana* and *Oryza sativa*.** The typical domains (HFD_POLE4-like, HFD_CHRAC1-like and CBFD_NFYB_HMF) are indicated by black solid lines.
